# Genome-Wide Analysis of the PHT Gene Family and Its Response to Mycorrhizal Symbiosis in Tomatoes under Phosphate Starvation Conditions

**DOI:** 10.3390/ijms241210246

**Published:** 2023-06-16

**Authors:** Wenjing Rui, Jing Ma, Ning Wei, Xiaoya Zhu, Zhifang Li

**Affiliations:** Beijing Key Laboratory of Growth and Developmental Regulation for Protected Vegetable Crops, Department of Vegetable Science, College of Horticulture, China Agricultural University (CAU), Yuanmingyuan Xilu 2, Haidian District, Beijing 100193, China

**Keywords:** arbuscular mycorrhizal fungi, phosphate transporters (PHT), genome-wide analysis, expression analysis, tomato

## Abstract

Phosphate is one of the essential mineral nutrients. Phosphate transporter genes (PHTs) play an important role in Pi acquisition and homeostasis in tomato plants. However, basic biological information on PHT genes and their responses of symbiosis with arbuscular mycorrhizal in the genome remains largely unknown. We analyzed the physiological changes and PHT gene expression in tomatoes (*Micro-Tom*) inoculated with arbuscular mycorrhizal (AM) fungi (*Funneliformis mosseae*) under different phosphate conditions (P1: 0 µM, P2: 25 µM, and P3: 200 µM Pi). Twenty-three PHT genes were identified in the tomato genomics database. Protein sequence alignment further divided the 23 PHT genes into three groups, with similar classifications of exons and introns. Good colonization of plants was observed under low phosphate conditions (25 µM Pi), and Pi stress and AM fungi significantly affected P and N accumulation and root morphological plasticity. Moreover, gene expression data showed that genes in the SlPHT1 (*SlPT3*, *SlPT4*, and *SlPT5*) gene family were upregulated by *Funneliformis mosseae* under all conditions, which indicated that these gene levels were significantly increased with AM fungi inoculation. None of the analyzed SlPHT genes in the SlPH2, SlPHT3, SlPHT4, and SlPHO gene families were changed at any Pi concentration. Our results indicate that inoculation with AM fungi mainly altered the expression of the PHT1 gene family. These results will lay a foundation for better understanding the molecular mechanisms of inorganic phosphate transport under AM fungi inoculation.

## 1. Introduction

Phosphorous (P) is important for plant growth and development, and it is an important component of signal transduction and associated cellular processes [1]. However, the Pi concentration is always very low (<10 µM) in soil because of its low solubility and uneven distribution [2,3]. Plants have developed a range of strategies to deal with Pi stress during their adaption to the environment. These strategies include the induction of Pi transporter genes, remobilization of internal Pi, root system architecture changes, and symbiosis with mycorrhizae [4,5]. Most terrestrial vascular plants have reciprocal relationships with arbuscular mycorrhizal (AM) fungi [6]. AM fungi extract Pi from a soil volume encompassing the entire “mycorrhizosphere” via their extraradical hyphae in soil areas distant from the root surface; Pi is then transported via the “mycorrhizal pathway” along fungal hyphae to the specialized symbiotic interfaces within the root cortex in exchange for plant-based sources of carbon [7,8].

Plants take up P from the soil in the form of Pi (H_2_PO_4_^−^). Pi uptake and transport in plants generally occur via phosphate transporters (PHTs) [9,10]. The yeast *PHO84* gene was the first protein identified with high affinity for PHT [11]. An increasing number of PHT families have been identified in a variety of plants, including Arabidopsis [12,13], tomato [14], maize [15], wheat [16,17], millet [18], camelina [19], and rice [20]. Plant PHTs are generally grouped into six phylogenetically distinct subfamilies, designated PHT1–PHT5 and PHO [21]. The plant PHT1 subfamily belongs to the Pi/H^+^ symporter family, which is a family member of the large major facilitator superfamily (MFS) [22]. PHT2, PHT3, and PHT4 localize between the cytoplasm and plastids, mitochondria, and Golgi membranes for energy metabolism; the PHT5 family plays a crucial role in vacuolar mediation of Pi storage and adaptation, and PHO has been reported in both Pi transport from root to shoot and the Pi deficiency response signal transduction cascade [23]. Arbuscular-mycorrhiza-induced Pi transporters belonging to the PHT1 family have been identified from several plant families, and their functions are associated with Pi uptake at the intraradical symbiotic interface, including *SlPT4* [14] in tomato, *StPT3* [24] in potato, *MtPT4* [25] in barrel medic, *OsPT11* [26] in rice, *LjPT4* [27] in *Lotus japonicus*, and *PhPT5* [28] in petunia. These AM-inducible PHTs are localized in peribranchial membranes and participate in the transport of P along the host’s mycorrhizal pathway.

The large majority of data regarding Pi transporters during AM symbiosis focus on the regulation of PHT1 type transporters. The PHT2 family exhibits a structure similar to that of PHT1, and PHT2 transporters have been shown to be involved in Pi allocation at the whole plant level and could thus be affected during AM symbiosis [29]. PHT3 is predicted to function via Pi: OH^_^ antiport and to catalyze Pi: Pi exchange between the matrix and the cytosol. Considering the known effects of AM fungi on plastid and mitochondrial biosynthetic pathways, PHT3 transporters are likely involved in channeling Pi transporters during AM symbiosis [30]. PHT4 also contributes to Pi transport within plant tissues, and PHT5 is involved in maintaining Pi homeostasis within plants [31]. Tomato (*Solanum lycopersicum*) is an important vegetable worldwide and a model plant for biological and genetic studies [32]. A previous study reported a genome-wide analysis, phylogenetic evolution, and expression patterns of PHT1 genes in tomato [14]. Eight members of this gene family have been identified in PHT1 (*PT1*–*PT8*). AM fungi interact with PHT1 transporters *SlPT3*, *SlPT4*, and *SlPT5* in tomato, and these genes are overexpressed in mycorrhizal roots [8]. Szentpeteri, et al. [33] suggested that *SlPT7* was also an AM-fungus-inducible phosphate transporter gene. *SlPT1*, *SlPT2*, and *SlPT6* were very significantly downregulated in mycorrhizal roots under low Pi supply conditions [14]. However, other PHTs have received less attention, and a comprehensive analysis of all PHT proteins (PHT1–PHT5 and PHO) in tomato is lacking. On the other hand, a previous study only considered the expression of PHTs in mycorrhizae and their regulatory mechanisms in mycorrhizal roots under one level of phosphate conditions.

The present study used bioinformatics and experimental investigations to identify members of tomato PHTs, and their characteristics were investigated for their expression patterns in response to AM fungi colonization under different levels of phosphate (P1: 0 µM, P2: 25 µM, and P3: 200 µM Pi). Several members of the PHT gene family induced by AM fungi colonization were identified. A detailed identification and analysis of tomato PHTs will provide new approaches to improve tomato Pi uptake efficiency.

## 2. Results

### 2.1. Identification and Phylogenetic Classification of the SlPHT Gene Family

We searched for SlPHT domain sequences and identified 23 putative SlPHT genes (8 SlPHT1, 1 SlPHT2, 4 SlPHT3, 4 SlPHT4, and 6 SlPHO) from the tomato genome (Table 1). Each SlPHT was named according to its location on the chromosome, and *SlPT1*–*SlPT8* genes belonging to the SlPHT1 subfamily were previously reported [14]. There was considerable variation in the protein length, and the gene size of SlPHTs ranged from 324 to 792 bases. The minimum and maximum gene sizes were 1446 bp (*SlPHT3.3*) and 3394 bp (*SlPHO1.4*), respectively. The molecular weight (MW) ranged from 35.67 kDa (*SlPHT3.2*) to 92.27 kDa (*SlPHO1.3*) and the PI ranged from 6.72 (*SlPHT4.4*) to 9.41 (*SlPHT3.2*).

### 2.2. Gene Structure and Protein Domain Analysis of SlPHT Members

**Figure 1:**
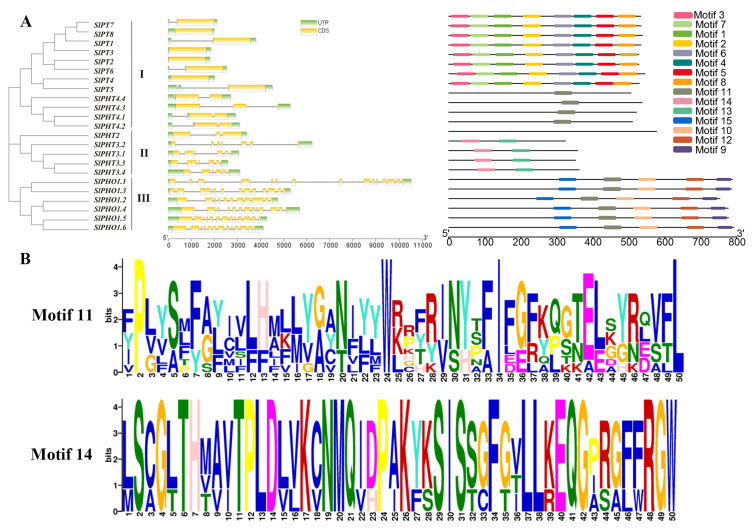
Phylogenetic relationship, gene structure, and motif analysis of the SlPHT genes. (**A**) The phylogenetic tree was constructed by the NJ method implemented in MEGA 11 software with 1000 bootstrap replicates. Exons are represented by green rectangles, and gray lines represent introns. The position of the SlPHT domains is indicated in yellow. The conserved motifs of typical members of each SlPHT subfamily are presented. (**B**) Visualization of motifs 11 and 14.

Exon–intron structure is an important evolutionary feature of genes and it provides a clue about the function of gene family members. To examine the structural diversity of the SlPHT genes in tomato, we evaluated the conserved exon–intron organization. There are three groups (I, II, and III) of SlPHT proteins, as defined by phylogenetic relationships (Figure 1A). The number of exons within SlPHT genes ranged between 1 and 15 according to gene structure analysis (Figure 1A). In group I, *SlPT1*–*SlPT8* genes only have 1 exon and *SlPHT4.1*–*SlPHT4.4* have 2 exons. Most SlPHT genes in group Ⅱ contained 6 exons, and four members in group Ⅲ contained 13 exons. *SlPHO1.1* had the highest number of exons. These results show that the intron/exon distribution patterns within the same subfamily were comparable.

A conservation motif distribution analysis was performed to further investigate tomato SlPHT structural features. We identified 15 conserved motifs, which were labelled motifs 1–15. Most members of the SlPHT1 gene family exhibited motifs 1–8 and several motifs were specific to only one or two PHT members. SlPHT2 did not exhibit any motifs, and SlPHT3 gene family included two motifs (motifs 13 and 14). Motif 11 was uniquely present in SlPHT4 and the SlPHO gene family, Proteins of the SlPHO gene family contained motifs 9, 10, 12, and 15, exclusively. A consensus sequence for each of these motifs is shown in Table S3. Genes with similar exon–intron structures and closer phylogenetic relationships exhibit a more similar conserved structural domain organization (Figure 1A).

### 2.3. Cis-Acting Element and Chromosomal Distribution

**Figure 2:**
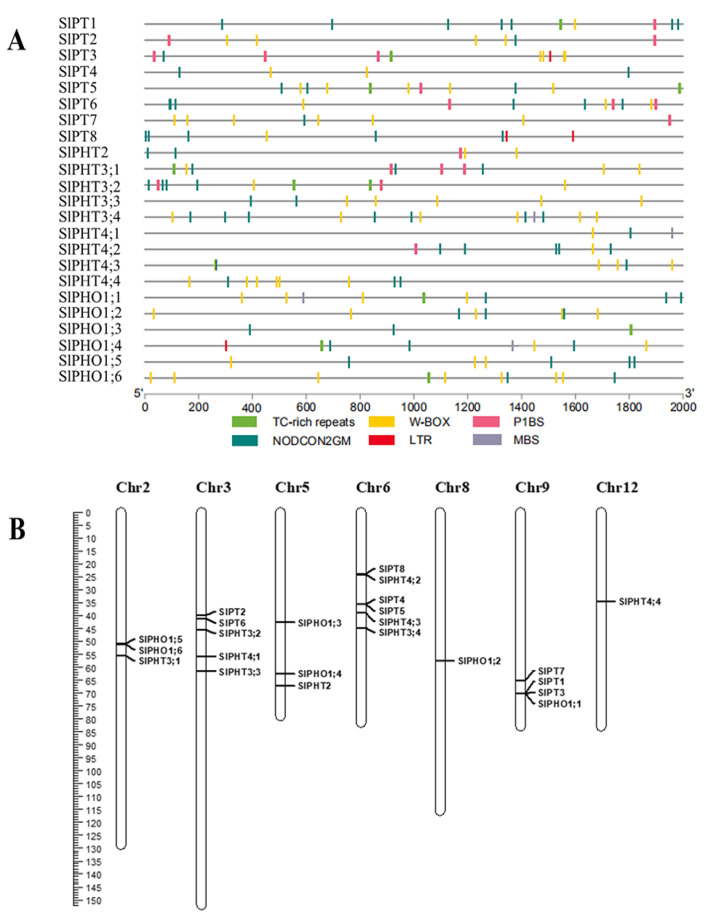
The main putative cis elements within 2 kb upstream promoter regions of SlPHT (**A**) and distribution of PHT genes on chromosomes in tomatoes (**B**).

We analyzed the cis-regulatory elements in the promoter sequences of the SlPHT genes to better understand their transcriptional regulation and potential functional roles. The 2 kb upstream region of the SlPHT promoter contained potential regulatory elements involved in AM and Pi responses. Ten identified SlPHT genes contained a P1BS element and most SlPHT genes contained TC-rich repeats and W-box, MBS, and LTR motifs. All of the SlPHT genes harboured the NODCON2GM and OSEROOTNO-DULE motifs (Figure 2A).

A mapchart was used to show the 23 SlPHT genes on the tomato chromosomes based on physical locations from a GFF3 file. The genes are distributed across seven chromosomes. Six SlPHT genes were located on chromosome 6, five SlPHT genes were located on chromosome 5, and chromosomes 8 and 12 each contained only one gene (Figure 2B).

### 2.4. Phylogenetic Analysis

**Figure 3:**
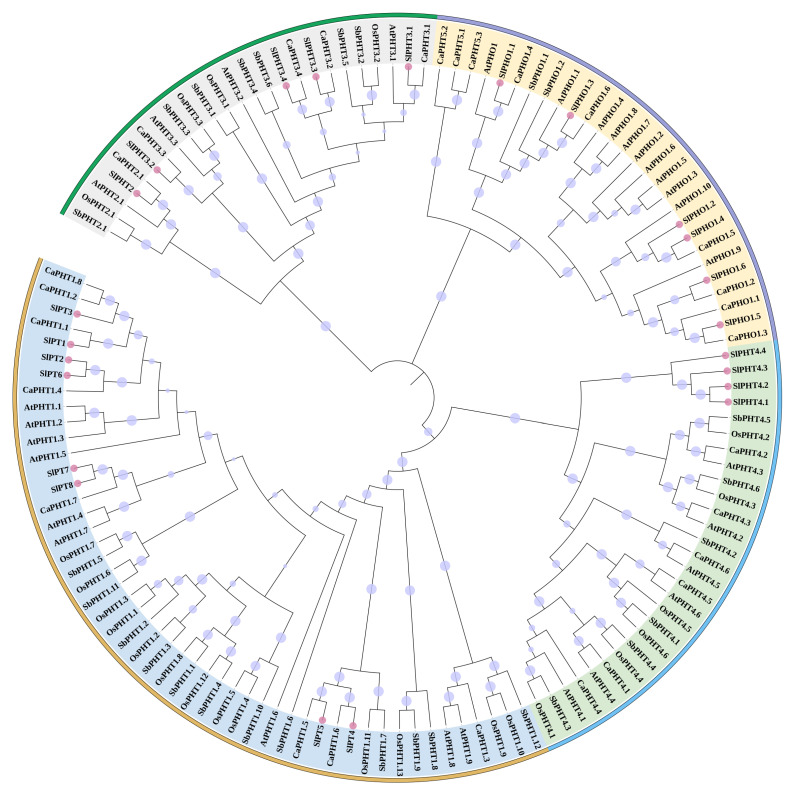
Phylogenetic tree showing the conserved domains of PHT proteins in *Solanum lycopersicum* and PHT genes from *Arabidopsis thaliana*, *Oryza sativa*, *Capsicum annuum*, and *Sorghum bicolor*. The phylogenetic tree was constructed by the NJ method implemented in MEGA 11 software with 1000 bootstrap replicates. Green: PHT2/PHT3 subfamily member; light purple: PHO1 subfamily member; blue: PHT4 subfamily member; yellow: PHT1 subfamily member. Purple circle: bootstrap values (17.85–100), Pink circle: PHT proteins in tomato.

To analyze the evolutionary relationship of the 23 genes, the phylogenetic relationships between tomato PHT genes and other homologues in plants were estimated using a phylogenetic tree. The 23 PHT genes identified in tomato belonged to the five PHT gene subfamilies known in plants: PHT1, PHT2, PHT3, PHT4, and PHO. Genes (eight genes) belonging to the PHT1 subfamily accounted for the greatest number of PHTs identified, while the PHT2 subfamily contained the lowest number of identified PHT genes (one gene) (Figure 3).

### 2.5. Mycorrhizal Colonization

**Figure 4:**
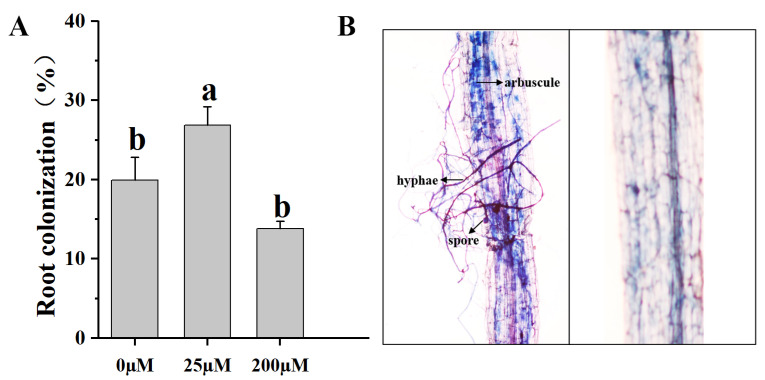
Impact of external Pi concentration on AM symbiosis in tomato roots. (**A**) Percentage of total mycorrhizal colonization by *Funneliformis mosseae* in tomato plants fertilized with different NaH_2_PO_4_ concentrations. (**B**) Typical structure of AM fungi in the root system of tomato, Scale bar = 100 μm. NM, non-mycorrhizal; AM, arbuscular mycorrhizal. Values are the mean ± SEs of three biological replicates from one pot (*n* = 3). Bars with the same letters above them are not significantly different at the *p* < 0.05 level.

To better understand the effects of Pi concentrations on AM symbiosis in tomato roots, we investigated mycorrhizal colonization. Five weeks after inoculation with *Funneliformis mosseae* under 25 μM Pi concentration, greater than 20% of tomato roots contained AM colonization structures, and none of the NM tomato roots contained AM colonization structures at any Pi concentration (Figure 4B). The 25 μM Pi concentration significantly increased the root AM fungi colonization rate compared with Pi concentrations of 0 μM and 200 μM (Figure 4A).

### 2.6. AM Fungal Colonization Promotes Tomato Growth and Nutrient Uptake

**Figure 5:**
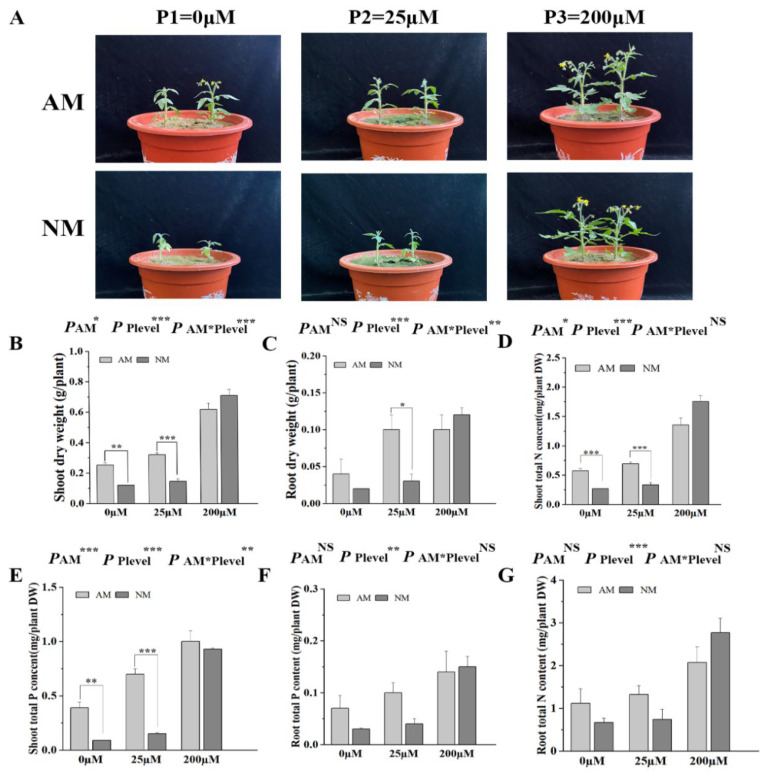
Effects of AM fungal colonization on tomato plant growth and P/N uptake under different concentrations of H_2_PO_4_^−^ supply. (**A**) The plants were watered with nutrient solution containing different concentrations of H_2_PO_4_^−^ weekly and harvested at 5 weeks post-inoculation (wpi) to assay the biomass (**B**,**C**), shoot total N/P content (**D**,**E**), and root total N/P (**F**,**G**) contents. NM, non-mycorrhizal; AM, arbuscular mycorrhizal. Values are the means ± SEs of three biological replicates from one pot (*n* = 3). The asterisks indicate significant differences. * *p* < 0.05, ** *p* < 0.01, *** *p* < 0.001.

After 5 weeks of growth, shoot and root dry weight inoculated with AM fungi increased significantly compared with the non-inoculated treatment under 25 μM Pi concentration (Figure 5B,C). The shoot total P and N contents of AM plants were significantly increased compared with those of NM plants under 0 μM and 25 μM Pi concentrations (Figure 5C,D). No significant difference was observed in the plant biomass and N and P uptake between the inoculated treatment and non-inoculated treatment under 200 μM Pi concentration. Two-way ANOVA revealed that P treatment and AM fungi inoculation more significantly affected the shoot dry weight, root dry weight, and shoot total P content than the shoot total N content, root total P content, and root total N content.

### 2.7. AM Fungal Colonization Promotes Tomato Root Morphological Plasticity

**Figure 6:**
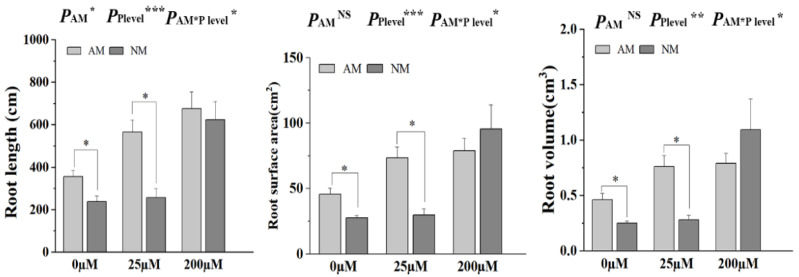
Effects of AM fungal colonization on tomato root morphological plasticity under different concentrations of H_2_PO_4_^−^ supply. NM, non-mycorrhizal; AM, arbuscular mycorrhizal. Values are the means ± SEs of three biological replicates from one pot (*n* = 3). The asterisks indicate significant differences. * *p* < 0.05, ** *p* < 0.01, *** *p* < 0.001.

The tomato root morphological plasticity (root length, root surface, and root volume) was significantly increased after AM fungi inoculation compared with the non-inoculated treatment under Pi concentrations of 0 μM and 25 μM. However, no significant difference was observed in the root morphological plasticity between the inoculated treatment and non-inoculated treatment under 200 μM Pi concentration (Figure 6). Two-way ANOVA revealed that P treatment and AM fungi inoculation significantly affected all root morphological plasticities.

### 2.8. Orthogonal Partial Least-Squares Discrimination Analysis (OPLS-DA) of Physiological Indicators

**Figure 7:**
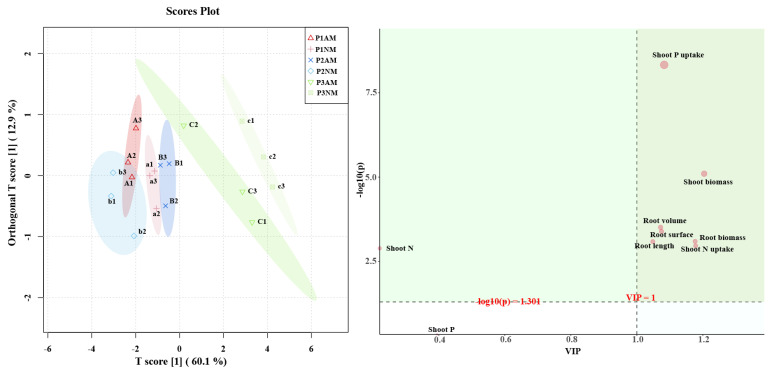
Orthogonal partial least-squares discrimination analysis results of physiological indices of tomato under different P and inoculation conditions. Red (triangles), P1AM (P1 = 0 μM Pi); orange (+), P1NM (P1 = 0 μM Pi); blue (×), P2AM (P2 = 25 μM Pi); blue (diamonds), P2NM (P2 = 25 μM Pi); green (inverted triangles), P3AM (P3 = 200 μM Pi); green (checked boxes), P3NM (P3 = 200 μM Pi).

According to the OPLS-DA results, the AM and NM plants were clearly distinguished under all conditions. This result suggests that inoculation had a great impact on the physiological indicators of tomato compared with non-inoculated tomato under all conditions. The main physiological indices that produced differences in tomato were shoot P uptake, shoot biomass, shoot N uptake, and root morphological plasticity (Figure 7, VIP > 1 and FDR < 0.05). Our findings demonstrated that the influence of Pi status on the development of AM fungi affected tomato growth.

### 2.9. The Expression Patterns of SlPHT Genes in Response to AM and Pi Concentrations

**Figure 8:**
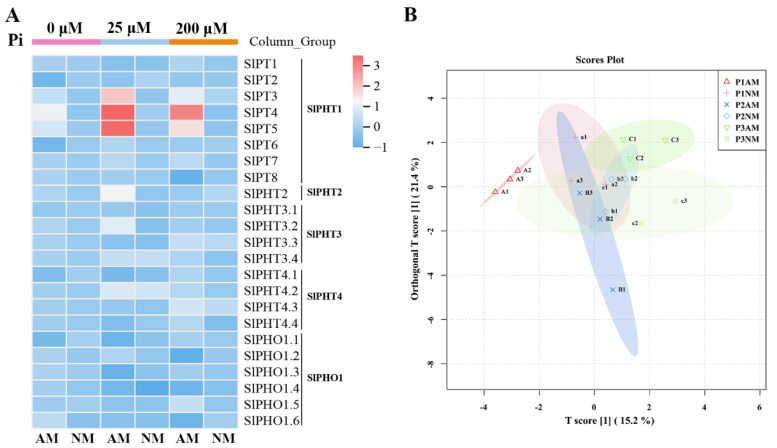
Heatmap analysis and OPLS-DA results of SlPHT gene expression in tomato roots under different P and inoculation conditions. (**A**) Transcripts of SlPHT in tomato roots of mycorrhizal (AM) and non-mycorrhizal (NM) plants. The color scale represents the variation in the expression of SlPHT, from high (red) to low (blue) contents. (**B**) Red (triangles), P1AM (P1 = 0 μM Pi); orange (+), P1NM (P1 = 0 μM Pi); blue (×), P2AM (P2 = 25 μM Pi); blue (diamonds), P2NM (P2 = 25 μM Pi); green (inverted triangles), P3AM (P3 = 200 μM Pi); green (checked boxes), P3NM (P3 = 200 μM Pi).

To better understand the possible function of tomato PHT genes, transcript levels of SlPHT genes were investigated in roots inoculated with AM under different concentrations of Pi. The genes exhibited different expression patterns under AM fungal colonization and Pi stress. These clusters and subclusters are represented by a heatmap (Figure 8A). Genes in SlPHT1 (*SlPT3*, *SlPT4*, and *SlPT5*) were upregulated by *Funneliformis mosseae* under all conditions, which indicated that these gene levels were significantly increased with AM fungi inoculation. There was a noticeable difference in the expression of *SlPT3*, *SlPT4*, and *SlPT5* in AM-induced roots compared with non-inoculated roots. *SlPT2* was downregulated by AM fungi under Pi concentrations of 0 μM and 200 μM. None of the analyzed SlPHT genes in the SlPH2, SlPHT3, SlPHT4, and SlPHO gene families were upregulated at any Pi concentration. The present study provides a valuable description of SlPHT gene expression regulation during AM fungal colonization and Pi stress. OPLS-DA (Figure 8B) data showed that AM and NM plants were clearly distinguished under all conditions. The main genes differentially expressed in roots in response to mycorrhizal inoculation mainly belonged to the PHT1 gene family. Taken together, the results indicate that inoculation with AM fungi mainly altered the expression of the PHT1 gene family.

## 3. Discussion

### 3.1. Identification and Characterization of SlPHT Genes in Tomato

Nucleic acids, phospholipids, and ATP are all derived from P, which is biologically active and involved in a wide range of processes, such as energy transfer, toleration of stress, and cell signaling [21]. Pi transfer from fungi to plants was established using ^32^P- or ^33^P-labelled substrates [34,35]. Pi is acquired by extraradical mycelia from the soil, transported to the roots, and transferred to the cells of the plant in a symbiotic process. PHT genes are a key component in this process [36]. The PHT gene was identified in a variety of plant species [37]. The transcript levels of many PHT1 transporters proteins decrease with increasing Pi levels, and the expression of a small subgroup of PHT1 transporter proteins in AM symbiosis is actually induced in mycorrhizal roots [30]. *OsPT11* is a mycorrhizal-specific PHT1 member in rice; owing to gene duplication, there are two orthologues of *OsPT11* in tomato: *SlPT4* and *SlPT5*. *SlPT4* is exclusively expressed during symbiosis, unlike *OsPT11*, and it is dispensable for symbiotic P uptake [38]. The expression patterns of *SlPT3* and *SlPT5* are relatively similar [39]. Past research on AMF-induced P transporters have mainly focused on the PHT1 family, and there are few studies on the other PHT families. In this research, we analyzed the physiological changes and PHT gene expression in tomatoes inoculated with AM fungi under different phosphate conditions. These results will lay a foundation for better understanding the molecular mechanisms of inorganic phosphate transport under AM fungi inoculation in tomato.

We reported the first systematic analysis of the SlPHT genes using bioinformatics tools and expression profiling analysis. We also performed a bioinformatic characterization of the SlPHT genes, including gene structure, chromosomal distribution, phylogenetic analysis, and cis-element analyses (Figure 1, Figure 2 and Figure 3). The tomato genome exhibits great complexity, which was evidenced by the different lengths of the nucleotide sequences between the 23 SlPHT genes [40]. The identified number of SlPHT genes in tomato was relatively greater than tair [41], but less than that of *Capsicum annuum* [21]. Phylogenetic analysis of SlPHTs revealed similar evolutionary divergences that reflected different biochemical and functional properties. The SlPHT1 family in tomato had the most genes, which is similar to sorghum [42], potato [43], and capsicum [21], and was located on tomato chr3, chr6, and chr9. Potatoes [43], pepper [21], and tomato contain a single gene from the PHT2 family. Previous studies identified three AtPHT3 genes from tair [44] and two StPHT3 genes from potato [43], whereas our study has identified four SlPHT3 genes located on tomato chr3 and chr6. Tair and rice contain six PHT4 genes, but tomato only had four SlPHT4 genes, located on chr3, chr6, and chr12. The present study identified six SlPHO genes. A common motif composition was shared by the most closely related members of the phylogenetic tree. Motifs 1–8 were widely distributed in the SlPHT1 family. Notably, SlPHT2 did not contain conserved motifs. Motifs 13 and 14 were specific to SlPHT3 and motif 11 was uniquely present in SlPHT4 and the SlPHO family, These conserved motifs may play a structural role in active proteins [45].

Transcriptional regulation may be investigated by analyzing the cis elements of genes using their transcription factors [46]. Many plants respond to stress by exhibiting TC-rich and W-box motifs [21]. SlPHT genes also contain these cis elements, and the present study demonstrated that these elements played an important role in controlling transcription when stressed. Numerous AM-inducible Pi transporter genes contain the P1BS and PHR1 cis-regulatory elements (Zhang et al. 2019). Our results showed that *SlPT3* and *SlPT5* contained a P1BS element. However, the mycorrhizal-specific gene *SlPT4* did not. These results suggest that the possession of P1BS itself does not provide information on whether the gene is related to AM symbiosis, as non-responding genes also contained P1BS, thus there is another element playing a role in the AM-symbiosis-related PHT expression. NODCON2GM(CTCTT) and OSEROOTNODULE (AAAGAT) were particularly active in cells because the two consensus sequence motifs were colonized by AM fungi in root nodules and arbuscular-containing cells (Vieweg et al. 2004). Our results suggest that all of the SlPHT genes harbored NODCON2GM and OSEROOTNODULE motifs (Figure 2).

### 3.2. Choice and Validation of P Supply Conditions Affecting AM Symbiosis

The mycorrhizal rate is determined by the plant and fungal genotype and by biotic and abiotic environmental factors such as nutrient availability [47]. Numerous studies investigated the effects of Pi on plant growth and AM symbiosis at a local and systemic level and throughout symbiosis. These studies demonstrated that ambient Pi availability and the Pi transporter within the plant influenced AM development [21,30,48,49,50]. Although high Pi concentrations generally inhibit AM symbiosis, this effect also depends on the inoculation method, fertilization conditions, the AM fungi, and the plant species. Breuillin et al. [51] indicated a good level of inhibition of AM symbiosis in pea, with only 7.5 mM Pi. Balzergue et al. [52] showed that 10 mM Pi was needed to achieve the same effect in petunia. Our results showed that root colonization was inhibited by increased P supply to the micro-tomato variety Micro-Tom, and root colonization was already very low under a concentration of 200 μM Pi in the nutrient solution (Figure 4). However, the effect of 1.3 mM P on mycorrhizal colonization was not significant in *M. truncatula* [48]. P deficiencies had a cumulative effect in AM formation, and tomatoes produced similar results in previous studies [53,54]. We further determined that Pi concentrations played important roles in plant symbiosis with AM fungi.

AM fungi inoculation improved plant growth and altered plant physiology and morphology [55,56]. Comparisons of different parameters between NM and AM plants showed that mycorrhizal symbiosis significantly increased the growth of tomato plants when P input was low or absent (0 and 25 µM) (Figure 5). The present study provides strong evidence that mycorrhizal association plays an important role in tomato survival under Pi stress [57]. We also found that N and P increased in tomatoes inoculated with AM fungi, which is consistent with a previous study [58]. Inoculation with AM fungus rapidly expanded the root morphological plasticity of the plants and significantly changed the root morphological plasticity [59,60]. AM symbiosis with 0 and 25 µM NaH_2_PO_4_ promoted the root surface, root length, and root volume in our study (Figure 6).

Mycorrhizal plants compensate for P deficiency by using the mycorrhizal P uptake pathway, and similar findings were noted in *P. hybrida* [61] and *M. truncatula* [62].

### 3.3. SlPHT Responses to Pi Availability and AM Fungi Inoculation

Numerous studies reported the modulation of gene expression via fungus inoculation and Pi stress. These specialized expressions of these genes reveal the divergency of regulatory components required to regulate Pi absorption and AM symbiosis. We measured the transcript level of 23 SlPHTs in AM-inoculated tomato roots under different Pi concentrations. The results revealed major differences in the expression patterns of 23 SlPHTs in response to symbiotic AM fungi. Many PHT genes are expressed significantly in the roots of several different plants, which suggest that these genes function in the capture and uptake of Pi [63,64]. *SlPT3*, *SlPT4,* and *SlPT5* were highly expressed in tomato roots inoculated with AM fungi in low phosphate conditions, as previously reported [14]. We found that *SlPT2* was suppressed in AM-inoculated tomato roots, which suggests that *SlPT2* may be race-specific (Figure 8). Consistent with previous research, AM colonization significantly affected plant Pi uptake, including an upregulation of AM-inducible Pi transporters and downregulation of Pi transporter genes involved in the direct Pi pathway [16]. However, they are mainly genes belonging to the PHT1 family of high-affinity phosphate transporter protein genes. Although most of the data on Pi transport during AM symbiosis focus on the regulation of the PHT1 type of transporter, other components of the Pi transport network are expected to play an active role in the reorganization of Pi fluxes in plants.

Members of the PHT2 family have been shown to be involved in Pi allocation at the plant-wide level [29] and thus may be affected during AM symbiosis. In our results, PHT2 gene expression remained unchanged after inoculation with AM fungi, as previous reported; to date, no PHT2 members have been found to respond to Pi levels or AM symbiosis [65,66,67]. Furthermore, altered expression of *SOLtuPht2;1* does not affect the expression levels of Pi transporters expressed in the same cells as PHT1, raising the question of whether these two transporter families are co-regulated at the transcriptional level. Nevertheless, the lack of altered transcriptional regulation does not preclude a role for this family in regulating symbiotic Pi allocation. Further evaluation of the various functions of the PHT2 transporter may lead to novel findings involving the reorganization of Pi fluxes that occur in plants as a result of AM symbiosis. In Arabidopsis, overexpression of *MicroRNA399b* down-regulates *PHT2;1* and has different effects on *Pht3;2* and *Pht3;3*, depending on the Pi status [68]. Interestingly, it is not known whether the *MicroRNA399* family plays a role in the regulation of AM co-occurring transporters and Pi signaling pathways.

There is little information on the possible involvement of the PHT3, PHT4, and PHO gene families in the reorganization of Pi transport in response to AM symbiosis. In our results, these gene expressions remained unchanged after inoculation with AM fungi. Considering the known effects of AM fungi on plastid and mitochondrial biosynthetic pathways, these transporters may be involved in Pi transport channels during AM symbiosis [69]. Many studies have also described the direct effect of fluctuations in Pi levels on the regulation of the biosynthetic pathway. The study of Pi transporters is a relatively new area of research and the involvement of these proteins in AM symbiosis has not been investigated. Therefore, their PHT3, PHT4, and PHO gene families’ involvement in AM symbiosis is unknown, but they are strong candidates to play a role in Pi redistribution during this symbiosis.

## 4. Materials and Methods

### 4.1. Plant Material and AM Fungi Inoculation

First, tomato (*Solanum lycopersicum* cv. Micro-Tom) seeds were sterilized with NaClO (5%) for 10 min, washed 2–3 times with sterilized distilled water, and then spread on sterile filter paper for 48 h in an incubator. Afterwards, sprouted seeds were germinated in autoclaved sand/vermiculite (1:1, *v*/*v*) irrigated with the following nutrient solution: 24.5 µM Fe^2+^, 22.98 µM BO_3_^3−^, 10 µM Mn^2+^, 0.66 µM Zn^2+^, 0.32 µM Cu^2+^, 0.07 µM MoO_4_^2−^, 1 mM Pi, 7.5 mM NO_3_^−^, 2.5 mM K^+^, 2.5 mM Ga^2+^, 1 mM Mg^2+^, and 1 mM SO_4_^2−^.

When the second true leaf was expanded, two uniform tomato seedlings were transplanted into a pot (3 L volume). The substrate was sterilized (121 °C, 1 h) river sand after rinsing with clean water 2–3 times. The selected sand contained 19 mg/kg total K, 0.08 mg/kg total P, a pH of 7.20, and an electrical conductivity (EC) of 0.12 mS/cm. Each pot was inoculated with 40 g of *Funneliformis mosseae* inoculum (a mixture of hyphae, approximately 100 spores, colonized root segments, and sand). *Funneliformis mosseae* belongs to species of Glomeromycota. Then, 40 g of autoclaved (121 °C, 1 h) inoculum and 10 mL of filtered washing of AM fungal inoculum was added to the controls (non-mycorrhizal plants). The microbial wash was carried out by filtering (<20 µm pore size) 200 mL of suspension prepared from 40 g of AM fungal inoculum.

### 4.2. Experimental Design

The experiments consisted of three P levels (P1:0 µM, P2: 25 µM, and P3: 200 µM Pi) (Supplementary Table S1) and two inoculation treatments (AM: with *Funneliformis mosseae* inoculation; NM: without *Funneliformis mosseae* inoculation). The experiment comprised six replicates for each treatment. The seedlings were watered with 400 mL 1/2 modified Hoagland’s solution at different P concentrations (pH 5.8) once a week (Supplementary Table S1). The tomato was grown in a greenhouse with 28 °C daytime and 18 °C nighttime temperatures. All of the groups were at 70%–80% field capacity.

At 5 weeks post-inoculation, the plants were harvested for collection of the root and shoot samples. Shoots and roots were washed with deionized water. Two parts of the roots were identified: the first part was used to examine nutrient concentration, root morphological plasticity, and mycorrhizal colonization, and an additional part was stored at −80 °C to isolate total RNA. Shoot and root samples were dried at 70 °C, weighed with the dry weight calculated, and then used to examine nutrient concentration.

### 4.3. Identification of Phosphate Transporter (PHT) Genes in the Tomato Genome

The genome information of tomato (GCF00188115.4) and SlPHT protein family domain sequences were downloaded from NCBI (https://www.ncbi.nlm.nih.gov accessed on 20 January 2023) and Pfam (http://pfam.xfam.org/ accessed on 20 January 2023), respectively [21]. Then, HMMER 3.0 windows were used to perform hidden Markov model (HMM) profiling on tomato protein sequences with an e-value <0.05 as a cut-off parameter, and duplicate sequences were removed manually. Furthermore, all candidate SlPHTs were analyzed by means of SMART (http://smart.embl.de/ accessed on 20 January 2023) in the genomic mode and conserved domain database. The physicochemical properties of SlPHT proteins were analyzed with Protoparam (https://web.expasy.org/protparam/ accessed on 22 January 2023).

### 4.4. Conserved Motif, Gene Structure, and Chromosomal Mapping

MEME (https://meme-suite.org/meme/ accessed on 25 January 2023) (a maximum of 15 motifs per sequence; sequences with zero or one occurrence) was used for motif analysis to identify conserved motifs. We determined the structure of the SlPHT gene by comparing the coding sequence to its genomic sequence using TBtools [70], and then citrus SlPHT protein motifs, conserved domains, and gene structure. Every SlPHT gene was matched with the chromosomes of tomato based on the genome annotations of tomato. Mapchart was used to draft the map.

### 4.5. Phylogenetic and Cis-Element Analysis

Multiple sequence alignment was carried out using ClustalW with the default settings [71]. We constructed the phylogenetic tree with neighbor-joining and 1000 bootstrap replicates in the pairwise gap deletion mode in MEGA7, and then annotated and visualized it with iTOL (https://itol.embl.de/ accessed on 28 January 2023). The upstream region of the selected SlPHT (2000 bp upstream of the translation initiation codon) was used to identify putative cis-acting elements using PlantCARE https://bioinformatics.psb.ugent.be/webtools/plantcare/html/ accessed on 30 January 2023 and PLACE https://www.dna.affrc.go.jp/PLACE/?action=newplace accessed on 30 January 2023 to search the cis-acting regulatory elements. TBtools was used to visualize the results [70,72].

### 4.6. Real-Time Quantitative Polymerase Chain Reaction (RT qPCR)

Total RNA was extracted from root samples of three biological replicates from each treatment. The Total RNA Isolation System (Waryoung, Beijing, China) was used to extract RNA from tomato roots (100 mg). First-strand cDNA was synthesized with a FastQuant RT Kit (Tiangen, Beijing, China) following the manufacturer’s instructions using sample cDNA. RT qPCR analysis was performed using SYBR Premix Ex Taq Mix (Takara, Shiga, Japan) with an Applied Biosystems QuantStudio 6 real-time PCR system. The comparative ΔΔCt method was used to calculate relative gene expression levels. UBIquitin and EF were used as the internal reference genes. Three independent experiments were conducted. The gene-specific primers used for RT qPCR are listed in Table S2.

### 4.7. Mycorrhizal Colonization Rates

Trypan blue was used to stain roots following the Phillips and Hayman [73] method. The gridline intersection method was used to calculate mycorrhizal colonization under a light microscope. The specific methods are as follows: first, fresh roots were placed in 10% KOH and boiled for 15 min; second, the roots were immersed in 1% HCl, stained with 0.05% trypan blue, and boiled for 5 min. For AM fungal structures, intersections of at least 100 lines per root sample were scored.

### 4.8. Determination of the Concentrations of N and P and Root System Parameters

Shoot and root nutrient content analysis and root morphological plasticity were measured in three biological replicates from each treatment. Here, 98% H_2_SO_4_ and 30% H_2_O_2_ were used for digestion of dried shoots and roots. A molybdate blue colorimetric method was used to calculate total P content; Kjeldahl’s method was used to calculate total N [74]. Root morphological plasticity was measured using a root scanner (Epson perfection, Nagano, Japan) to scan fresh roots and then using WinRHIZO (Regent Instrument Inc., Sainte Foy, QC, Canada). The scanned images were analyzed according to the manufacturer’s protocol.

### 4.9. Statistical Analyses

All data were analyzed by SPSS 20.0 software. Following an analysis of variance, Fisher’s least-significant difference (LSD) test was used to calculate the difference among treatments; the effects of phosphate and inoculation or their interaction were calculated by two-way ANOVA. Correlation heatmap analysis and orthogonal partial least-squares discrimination analysis (OPLS-DA) were performed using the Wekemo Bioinformatics cloud (https://www.bioincloud.techaccessed on 10 February 2023).

## 5. Conclusions

In the present study, we identified 23 SlPHT genes, including 8 SlPHT1, 1 SlPHT2, 4 SlPHT3, 4 SlPHT4, and 6 SlPHO genes, in tomato plants and characterized their physiological and biochemical properties, evolutionary relationships, gene structures, and conserved motifs. Protein sequence alignment divided the 23 PHT genes into three groups with similar classifications of exons and introns. Gene expression data showed that genes in the SlPHT1 (*SlPT3*, *SlPT4*, and *SlPT5*) gene family were upregulated in tomato roots inoculated with AM fungi. None of the SlPHT genes in the SlPH2, SlPHT3, SlPHT4, and SlPHO gene families were changed when inoculated with AM fungi. These results indicate that inoculation with AM fungi mainly altered the expression of the PHT1 gene family. These results lay a foundation for better understanding of the molecular mechanisms of inorganic phosphate transport under AM fungi inoculation.

## Figures and Tables

**Table 1:** Tomato PHT gene information.

Gene Name	Accession Number	Location	ORF Length (bp)	Size (bp)	Molecular Weight	PI
*SlPT1*	NP_001234361.2	9:70098389_70102955	538	2002	58,699.54	8.51
*SlPT2*	NP_001234043.1	3:398936_401108	528	1826	57,762.29	8.61
*SlPT3*	NP_001318089.1	9:70109008_70111237	534	1867	58,536.25	8.67
*SlPT4*	NP_001234674.2	6:35656579_35659001	545	2019	60,502.44	8.49
*SlPT5*	XP_004240951.1	6:35659554_35664968	529	2384	59,037.82	8.73
*SlPT6*	XP_004234039.1	3:411956_415011	528	1857	57,762.29	8.61
*SlPT7*	XP_004247235.1	9:65061109_65063652	533	1793	58,300.12	8.90
*SlPT8*	XP_004240760.1	6:23920599_23923011	534	2011	58,486.39	8.90
*SlPHT2*	XP_004239128.1	5:6724597_6728688	577	2180	61,077.15	9.28
*SlPHT3.1*	NP_001266267.1	2:55614122_55617796	358	1593	38,440.82	9.22
*SlPHT3.2*	XP_004234502.1	3:4560513_4568003	324	1497	35,672.47	9.41
*SlPHT3.3*	XP_004235310.1	3:61601724_61604831	352	1446	37,656.50	9.18
*SlPHT3.4*	XP_004242142.1	6:44858180_44861909	362	2213	38,663.85	9.25
*SlPHT4.1*	XP_004235159.1	3:55746222_55749729	521	2176	56,581.50	6.40
*SlPHT4.2*	XP_004240757.1	6:24109636_24113354	510	2109	55,395.36	8.50
*SlPHT4.3*	XP_004242279.1	6:39021711_39028059	537	2465	58,269.86	8.36
*SlPHT4.4*	XP_010313844.1	12:3467625_3470879	506	2183	54,221.91	6.72
*SlPHO1.1*	XP_004247746.1	9:70338431_70351065	788	2766	90,815.17	9.21
*SlPHO1.2*	XP_019070605.2	8:57407221_57412919	753	2950	87,205.56	9.10
*SlPHO1.3*	XP_004238979.1	5:4267987_4274342	786	2646	92,036.01	9.17
*SlPHO1.4*	XP_010320919.1	5:6256934_6264807	776	3394	91,158.92	9.36
*SlPHO1.5*	XP_004232209.1	2:51014310_51019443	777	3063	89,979.97	9.35
*SlPHO1.6*	XP_004232205.1	2:51026560_51031505	792	2917	92,266.21	8.67

## Data Availability

The data used to support the findings of this study are available from the author upon request.

## Supplementary Materials

The following supporting information can be downloaded at: https://www.mdpi.com/article/10.3390/ijms241210246/s1.
