# Supplementary material for: Genome-Wide Analysis of the PHT Gene Family and Its Response to Mycorrhizal Symbiosis in Tomatoes under Phosphate Starvation Conditions
Source: Int J Mol Sci. 2023 Jun 16;24(12):10246. doi: 10.3390/ijms241210246 (PMC10298980; doi:10.3390/ijms241210246)
Supplement: Supplementary file 1 [file ijms-24-10246-s001.zip › ijms-2366036-supplementary.pdf]

Table S1 Nutrient solution composition used in this study

Macronutrient solution(μM)			
	0μM	25μM	200μM
$MgSO_4 \cdot 7H_2O$	1000	1000	1000
KNO_3	2500	2500	2500
$NaH_2PO_4 \cdot 2H_2O$	0	25	200
NaCl	1000	975	800
KCl	1000	1000	1000
$Ca\ (NO_3)_2 \cdot 4H_2O$	2500	2500	2500
Micronutrient solution (μM)			
EDTA-Fe	24.52		
H_3BO_3	22.98		
$MnSO_4 \cdot H_2O$	10.00		
$ZnSO_4 \cdot 7H_2O$	0.66		
$CuSO_4 \cdot 5H_2O$	0.32		
$(NH_4)_6Mo_7O_2 \cdot 4H_2O$	0.07		

Table S2 Primers of *SlPHT* genes used in the experiments.

	Primer sequence (5'→3')	
Gene name	Forward	Reverse
SlPT1	AGGGGAAGAGGAAACTGTAGCTG	ATACCACAAATTAACTCAAACTGCAT
SlPT2	GCCAGAGCCAAAAGGAAAATC	TGCAACAAACAAGCTTACACAATACA
SlPT3	TTGTGTTAGGTTGTGTGAATTTTCTT	AGCTCTTTGCACGATCTTAAATGAC
SlPT4	CGGGCAGAATGAGACACAGATG	TGAAGATAGAAAGCACAAGGCGTAGT
SlPT5	GCAGAACGAGACGCAGATGAA	TGCTGAATTTGATAAACTTGCCAA
SlPT6	GCCAGAGCCAAAAGGAAAATC	AAGAGTTGCATCAGTCATCACACA
SlPT7	AGGGGAAGTCACTGGAAGAGATG	GCAGCAATGACAGATAACCTAATACGT
SlPT8	AAGGGAAAACGAAGACTCAGCAC	AGGTTGAGGTAAAGAAACTATAGTGCT
SlPHT2	AGATTGCAACTTCCTGGGCA	CCGAGAAGTTTGTGCTGCAT
SlPHT3;1	CAACAATGCGAAGGGTGCAA	ATTCCCCATTGAGCACCAGT
SlPHT3;2	TCTAACCCCGCTGACAACAT	TCACAACTGGTCCAACAAGC
SlPHT3;3	GCCTGGATTTGCTAGAGGCT	TGTCGTCCCCATAGAGGTACA
SlPHT3;4	ATTGTGTCACACCCTGCTGA	AGACCGCGAGTACAAAGACC
SlPHT4;1	CACAACTGCTGTTTCGGCTG	CGCTCCAATTGAGCCAGTTC
SlPHT4;2	TCTCCCGGGAAAGAAGACGA	CCGGGATCTTCCATGCTTCT
SlPHT4;3	CATGGCCTATTGGTCCGCTT	CGACGTTCCATCCTCTCCAT
SlPHT4;4	TGGGTAGTGGGGTTTTGGTG	ACAAGGCCACCCAATAGACTG
SlPHO1;1	CAGTTATGGTTCTCGATCGTCT	GTCGAATAACTTCCAATGAGGC
SlPHO1;2	CCAACTTCTTCTTCAAGCGAAA	GATGATTTTGGACTGTCGGATG
SlPHO1;3	CTACAGGGTAACAGGGCTATTT	TGCTCATTCTCCAACCTGTAAT
SlPHO1;4	CGGTTGATGGGAAGCTTTGGA	CCAACTTTACGACTATCTTTCGCTT
SlPHO1;5	CCTTCAATTGGTATGCGTAGTG	CAAAATCTCTATTGGAGCAGGC
SlPHO1;6	AAAGTAGGGGAAGTTAAGGTGG	TGTGGTCTACCTGGACTACTAA
UBIquitin	TCGTAAGGAGTGCCCTAATGCTGA	CAATCGCCTCCAGCCTTGTTGTAA
EF	CTCCATTGGGTCGTTTTGCT	GGTCACCTTGGCACCAGTTG

Table S3 Fifty different motifs commonly observed in SlPHT proteins

Motif	Motif sequence	Width
1	MATLCFFRFWLGFGIGGDYPLSATIMSEYANKKTRGAFIAAVFAMQGFGI	50
2	VPQADYVWRIILMFGALPALLTYYWRMKMPETARYTALVAKBAKQAAADM	50
3	VLNALDTAKTQLYHFTAIVIAGMGFFTDAYDLFCISLVTKLLGRJYYHKP	50
4	QTLIALCSTVPGYWFTVAFIDKIGRFAIQLMGFFFMTVFMFAJAIPYHHW	50
5	YSLTFFFANFGPNATTFVVPAEIFPARLRSTCHGISAAAGKAGAMVGAFG	50
6	RHGLHLLGTASTWFLLDIAFYSQNLFQKDIFSAIGWIPKAKTMNALZEVY	50
7	PGSLPPNVSAAVNGVALVGTLAGQLFFGWLGDKMGRKKVYGMTLMJMVIC	50
8	KTDAGYPAGIGIKNSLIVLGCVNLLGMLFTFLVPESKGKSLEEMSRENEG	50
9	ACLEIJRRGMWNFFRLENEHLNNVGKYRAVKSVPLPFNYDD	41
10	PLNIIYRSSRFFFIRVIWRCLCAPLYKVTLPDFFLADQLTSQVQAIRSLZ	50
11	FPLYSLFAYIVLHMLLYGANIYYWKRYRINYSFIFGFKQGTELKYREVFL	50
12	WKVLVLVTSGIATVYNTYWDLVVDWGLLQRKSKNPWLRDKLLLPHKSV	48
13	CPFEAVKVRVQTQPGFARGLSDGLPKFVRAEGAAGLYKGLVPLWGRQIPY	50
14	LSCGLTHMAVTPLDLVKCNMQIDPAKYKSISSGFGVLLKEQGPRGFFRGW	50
15	LKTYSYLNLLAFSKILKKYDKITSRKASKSYLKVVDKSYLGSSDEVTKL	49
